# Involvement of the Akt signaling pathway in ER-α36/GRP94-mediated signaling in gastric cancer

**DOI:** 10.3892/ol.2014.2514

**Published:** 2014-09-09

**Authors:** ZHENGQI FU, HONGYAN ZHEN, FENG ZOU, XUMING WANG, YING CHEN, LIJIANG LIU

**Affiliations:** 1 Department of Pathology and Pathophysiology, School of Medicine, Jianghan University, Wuhan, Hubei 430056, P.R. China; 2 Jiangda Pathology Institute, Jianghan University, Wuhan, Hubei 430056, P.R. China

**Keywords:** gastric cancer, glucose-regulated protein 94, estrogen receptor-α36, Akt

## Abstract

Glucose-regulated protein 94 (GRP94) has been implicated in the promotion of tumor proliferation and metastasis. Previous studies have found that GRP94 is involved in the malignant growth of gastric carcinoma cells through estrogen receptor-α36 (ER-α36)-mediated estrogen signaling, but the underlying mechanism remains unclear. In the present study, we examined the expression levels of GRP94 and ER-α36 in tumor specimens from gastric cancer patients by immunohistochemistry, and found that both GRP94 and ER-α36 were highly expressed in the cytoplasms of gastric carcinoma cells. Furthermore, treatment with 17β-estradiol at a concentration of 10^−12^ M for 24 h increased the expression levels of GRP94 and ER-α36, and the phosphorylation levels of Akt at the Ser473 site (Ser473-Akt). In established SGC7901 gastric cancer cells with knockdown of ER-α36 expression, the levels of GRP94 and Ser473-Akt expression were significantly reduced. Thus, the Akt signaling pathway is a potentially important signaling pathway in ER-α36-GRP94-mediated gastric carcinogenesis.

## Introduction

Glucose-regulated protein 94 (GRP94) is a molecular chaperone in the endoplasmic reticulum that binds to misfolded proteins and unassembled complexes, with an important role in the maintenance of cellular homeostasis and the suppression of cell death in stress conditions. GRP94 is highly expressed in cancer tissues, and previous studies have shown that GRP94 is involved in tumor proliferation, metastasis, drug resistance and immunotherapy (1,2). In gastric carcinoma, GRP94 overexpression has been associated with increased tumor size, increased depth of invasion, lymphatic and venous invasion, and advanced stage (3). GRP94 cleavage, induced by honokiol through calpains, has been shown to induce apoptosis in human gastric cancer cells and reduce gastric tumor growth (4). However, the involvement of GRP94 in carcinogenesis has not been well established.

Gastric cancer is the fourth most common type of cancer and the second leading cause of cancer-related mortality worldwide (5,6). Epidemiological studies have demonstrated a global gastric cancer predominance in males. Tamoxifen, an antiestrogen agent, has been shown to accelerate tumor progression and increase the overall risk of gastric adenocarcinoma (7,8). These findings suggest an association between estrogen signaling and the pathogenesis of gastric cancer. Previous studies have found that estrogen receptor-α36 (ER-α36), a novel variant of ER-α, is highly expressed in human gastric cancer, and that ER-α36 expression levels were positively correlated with lymph node metastasis and GRP94 expression levels (3,9,10). However, the molecular mechanism by which ER-α36 functions through GRP94 in the pathogenesis of gastric cancer remains unclear.

In the present study, GRP94 and ER-α36 expression levels in gastric cancer samples were examined. To clarify the mechanism of GRP94 involvement in gastric carcinogenesis through ER-α36 signaling, SGC7901 human gastric adenocarcinoma cells were treated with 17β-estradiol (E2) and the expression levels of GRP94 and ER-α36, and the phosphorylation levels of Akt at the Ser473 site (Ser473-Akt) were measured. GRP94 and Ser473-Akt levels were also determined in established gastric cancer cells with knockdown of ER-α36 expression.

## Materials and methods

### Antibodies and chemicals

17β-E2 was purchased from Sigma-Aldrich (St. Louis, MO, USA). Polyclonal rabbit anti-mouse, anti-rat, anti-cow, anti-dog and anti-human GRP94 antibody was obtained from Abcam (Cambridge, UK). The monoclonal rabbit anti-human phospho-Akt at Ser473 (Ser473-Akt) antibody was purchased from Cell Signaling Technology, Inc. (Danvers, MA, USA). The rabbit anti-ER-α36 antibody was generated and characterized as previously described (11). The mouse anti-β-actin antibody was purchased from Santa Cruz Biotechnology, Inc. (Santa Cruz, CA, USA). Bicinchoninic acid protein detection kits, goat anti-mouse peroxidase-conjugated secondary antibody, chemiluminescence substrate kits and polyvinylidene difluoride (PVDF) membranes were obtained from Pierce Biotechnology, Inc. (Rockford, IL, USA). A third-generation SuperPicture immunohistochemistry (IHC) Detection kit was obtained from Invitrogen Life Technologies (Carlsbad, CA, USA). The radioimmunoprecipitation assay (RIPA) buffer and enhanced chemiluminescence reagents were obtained from the Beyotime Institute of Biotechnology (Shanghai, China).

### Cell culture

The SGC7901 human gastric adenocarcinoma cell line was obtained from the Cell Center of Basic Medicine, Chinese Academy of Medical Sciences (Beijing, China). SGC7901-low36, a gastric cancer cell line with knockdown of ER-α36 expression was established using the lentiviral small hairpin RNA method (11). The SGC7901 and SGC7901-low36 cells were cultured in RPMI-1640 medium (Gibco-BRL, Carlsbad, CA, USA) containing 10% fetal calf serum (FCS; HyClone Laboratories, Inc., Logan, UT, USA) at 37°C in a 5% CO_2_ atmosphere.

### 17β-E2 treatment

The cells were plated at a density of 1×10^6^ cells per 100-mm dish for 24 h, then washed with phosphate-buffered saline (PBS) and placed in phenol-red-free medium (Invitrogen Life Technologies, Carlsbad, CA, USA) with 5% charcoal-stripped FCS (HyClone Laboratories, Inc.) for 6 h, and then in 2% charcoal-stripped FCS (HyClone Laboratories, Inc.) for 24 h prior to the experiments. The cells were treated with 17β-E2 at a concentration of 10^−12^ M and vehicle (dehydrated alcohol) for 24 h.

### Gastric tumor samples

Paraffin-embedded tumor tissues from gastric cancer patients were obtained from the Jiangda Pathology Institute (Wuhan, China) between 2008 and 2009 following the approval of the Institutional Review Board. For immunohistochemical analysis, the tumor tissues were fixed in 10% neutral formalin, embedded in paraffin, and stained with hematoxylin and eosin. None of the patients had received any anticancer treatment prior to surgery. Written informed consent was obtained from all patients. This study was approved by the ethics committee of the School of Medicine, Jianghan University (Wuhan, China).

### Western blot analysis

Western blot analysis was performed according to methods previously established (12). Briefly, the cells were lysed and homogenized with RIPA buffer. The protein concentration was then estimated with the bicinchoninic acid kit according to the manufacturer’s instructions. The proteins were separated by 10% SDS-polyacrylamide gel electrophoresis and transferred to the PVDF membranes. The membranes were blocked with 5% non-fat milk dissolved in TBS-Tween-20 (containing 50 mm Tris HCl, pH 7.6, 150 mm NaCl and 0.2% Tween-20) for 1 h and probed with the primary antibodies (1;1,000) at 4°C overnight. The blots were then incubated with monoclonal goat anti-mouse or polyclonal goat anti-rabbit IgG conjugated to horseradish peroxidase (1:5,000) for 1 h at 37°C, and visualized with enhanced chemiluminescence. The blots were quantitatively analyzed by TotalLab analysis software (Nonlinear Dynamics Technical, NC, USA).

### Immunohistochemical assay

Immunohistochemical staining was conducted on 4-μm tumor sections via a ‘two-step’ assay (9). Briefly, the tissue slides were deparaffinized with xylene and rehydrated using a gradual alcohol series. Endogenous peroxidase activity was inhibited by incubating the slides in a 3% hydrogen peroxide/methanol buffer for 10 min. Antigen retrieval was performed by immersing the slides in EDTA buffer (pH 8.0) followed by boiling in a water bath for 25 min. The slides were subsequently rinsed in PBS and then incubated overnight at 4°C in a humidified chamber with either polyclonal anti-GRP94 antibodies at a dilution of 1:100 or polyclonal anti-ER-α36 antibodies at a dilution of 1:400. The slides were then incubated with the secondary antibody (horseradish peroxidase-conjugated polyclonal goat anti-rabbit Ig; 1:100; Invitrogen Life Technologies) for 30 min. Diaminobenzidine served as a chromagen and the slides were counterstained with hematoxylin. Images were observed and captured using an Olympus BX53 microscope and an Olympus DP72 digital camera, respectively (Olympus Corporation, Tokyo, Japan).

### Statistical analysis

SPSS 12.0 software (SPSS, Inc., Chicago, IL, USA) was used to conduct the analysis. All analyses were performed using Student’s t-test. P<0.05 was considered to indicate a statistically significant difference.

## Results

### GRP94 and ER-α36 are highly expressed in gastric tumors

**Figure 1:**
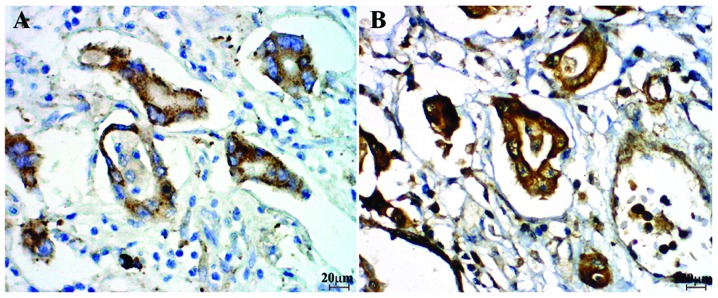
Immunohistochemical staining of gastric cancer tissues. (A) Glucose-regulated protein 94 and (B) estrogen receptor-α36 were highly expressed in the cytoplasm of gastric carcinoma cells (magnification, ×400).

The GRP94 and ER-α36 expression levels were examined in the specimens from gastric carcinoma patients using IHC assay. GRP94 and ER-α36 were highly expressed in the cytoplasms of gastric carcinoma cells (Fig. 1).

### E2 increases the protein expression levels of GRP94, ER-α36 and Ser473-Akt

**Figure 2:**
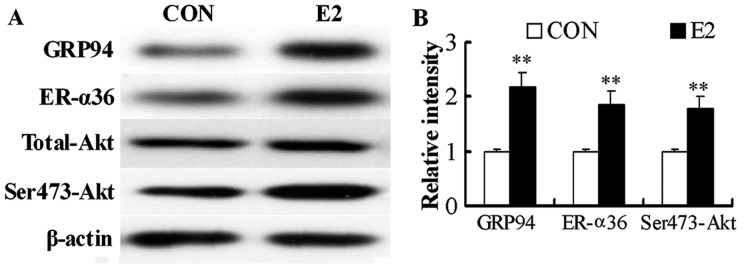
17β-estradiol (E2) treatment increases the protein expression levels of glucose-regulated protein (GRP)94, estrogen receptor (ER)-α36 and Ser473-Akt. SGC7901 human gastric adenocarcinoma cells were treated with 10^−12^ M E2 for 24 h. The addition of an equal volume of alcohol served as a control. The protein levels of GRP94, ER-α36 and Ser473-Akt were measured by western blotting, which was then quantitatively analyzed. (A) Western blotting and (B) quantitative analysis. The levels of GRP94, ER-α36 and total Akt were normalized to β-actin levels, and the levels of Ser473-Akt were normalized against total Akt levels. The data are presented as the mean ± SD of three independent experiments. ^**^P<0.01, vs. the control.

To investigate the involvement of GRP94 in gastric cancer estrogen signaling, the SGC7901 human gastric adenocarcinoma cells were treated with 10^−12^ M E2 for 24 h (14). Western blotting and quantitative analysis revealed a significant increase in Ser473-Akt, GRP94 and ER-α36 expression levels (P<0.01 for each; Fig. 2).

### ER-α36-mediated signaling regulates GRP94 expression through the Akt signaling pathway

**Figure 3:**
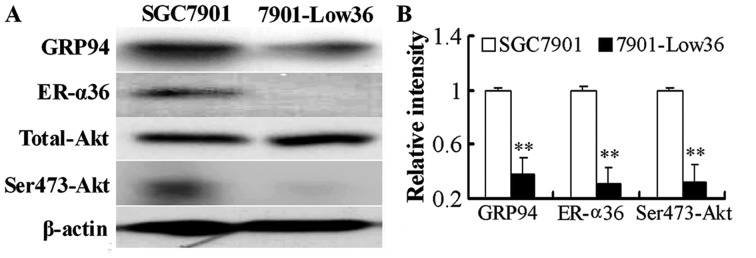
Western blot analysis of glucose-regulated protein (GRP)94, estrogen receptor (ER)-α36 and Ser473-Akt expression levels in SGC7901 gastric cancer cells. In SGC7901-Low36 (7901-Low36) cells (with knockdown of ER-α36 expression) the expression levels of GRP94 and Ser473-Akt were reduced, compared with SGC7901-control (SGC7901) cells transfected with an empty expression vector. The expression levels of GRP94, ER-α36 and Ser473-Akt were measured by western blotting, which was then quantitatively analyzed. (A) Western blotting and (B) quantitative analysis. The levels of GRP94, ER-α36 and total Akt were normalized against β-actin levels, and the levels of Ser473-Akt were normalized against total Akt levels. The data are expressed as the mean ± SD from three independent experiments. ^**^P<0.01, vs. SGC7901.

To further analyze the function of the Akt signaling pathway in ER-α36-GRP94 signaling, GRP94, ER-α36 and Ser473-Akt expression levels were examined in SGC7901-Low36 cells with knockdown of ER-α36 expression. Significant reductions in GRP94 and Ser473-Akt expression levels were observed in these cells compared with SGC7901 control cells transfected with an empty expression vector (P<0.01 for each; Fig. 3). These results suggest that the Akt signaling pathway is involved in ER-α36-mediated estrogen signaling through GRP94 in gastric carcinogenesis.

## Discussion

GRP94 is a chaperone in the endoplasmic reticulum that, under basal expression, controls normal physiological functions; however, GRP94 is also induced in pathological conditions, for example, hypoxia and nutrient deprivation (15). Under malignant conditions, GRP94 expression is upregulated and has been shown to be involved in the pathogenesis, growth, invasion and metastasis of gastric carcinoma (16). In our previous study, GRP94 was found to be highly expressed in human gastric adenocarcinoma tissues (3). The levels of GRP94 expression were significantly correlated with gender, tumor stage, lymph node metastasis and the expression levels of ER-α36, which is highly expressed in human gastric cancer and is involved in the malignant growth of gastric carcinoma cells (9,10). As estrogen is known to induce the expression of GRPs (3), this suggested that GRP94 may be involved in gastric carcinogenesis through ER-α36-mediated estrogen signaling.

Akt (also known as protein kinase B) is a serine/threonine protein kinase known to regulate the balance between cell survival and apoptosis (17). Activated Akt expression induces cell survival, whereas inhibition of Akt activity stimulates apoptosis (18,19). Studies have shown that the overexpression and/or activation of Akt occurs in gastric cancer (20,21), and that the phosphoinositide 3-kinase/Akt signaling pathway is important in the chemoresistance of gastric cancer cells against the cell death induced by etoposide and doxorubicin (22). These results suggest that the Akt signaling pathway is involved in tumor proliferation and drug resistance. In addition, E2 promptly activates the PI3K/Akt signaling pathway in Ishikawa cells in an ER-dependent and ER-independent manner in HEC-1A cells (23). E2 treatment has been demonstrated to increase the phosphorylation of Akt on Ser473 (Akt1), but does not activate Akt2 (on Ser474); furthermore, ER-α is required for Akt1 activation (24). Akt1 may increase ER-α protein levels, while simultaneously reducing transcriptional activity (25). In the present study, to clarify the mechanisms involving GRP94 in the pathogenesis of gastric cancer induced by the ER-α36 signaling pathway, SGC7901 cells were treated with E2; increased GRP94 and ER-α36 expression levels, as well as increased phosphorylation levels of Akt at Ser473, were observed. By contrast, in established gastric cancer cells with knockdown of ER-α36 expression, GRP94 and Ser473-Akt expression levels were significantly reduced. These results suggest that Akt may be a key downstream effector of ER-α36-GRP94-mediated signaling in gastric carcinogenesis.

In conclusion, in the present study, high expression levels of GRP94 and ER-α36 were identified in gastric cancer tissues. Furthermore, E2 treatment increased the expression levels of GRP94, ER-α36 and Ser473-Akt. In established gastric cancer cells with knockdown of ER-α36 expression, GRP94 and Ser473-Akt expression levels were significantly reduced. Thus, the Akt signaling pathway is a potentially important signaling pathway involved in ER-α36/GRP94-mediated signaling in gastric carcinogenesis.

## References

[1] Li J, Lee AS (2006). Stress induction of GRP78/BiP and its role in cancer. Curr Mol Med.

[2] Srivastava PK (2006). Therapeutic cancer vaccines. Curr Opin Immunol.

[3] Fu Z, Deng H, Wang X (2013). Involvement of ER-alpha36 in the malignant growth of gastric carcinoma cells is associated with GRP94 overexpression. Histopathology.

[4] Sheu ML, Liu SH, Lan KH (2007). Honokiol induces calpain-mediated glucose-regulated protein-94 cleavage and apoptosis in human gastric cancer cells and reduces tumor growth. PLoS One.

[5] Lambert R, Guilloux A, Oshima A (2002). Incidence and mortality from stomach cancer in Japan, Slovenia and the USA. Int J Cancer.

[6] Brenner H, Rothenbacher D, Arndt V (2009). Epidemiology of stomach cancer. Methods Mol Biol.

[7] Chandanos E, Lindblad M, Rubio CA (2008). Tamoxifen exposure in relation to gastric adenocarcinoma development. Eur J Cancer.

[8] Rutqvist LE, Johansson H, Signomklao T (1995). Adjuvant tamoxifen therapy for early stage breast cancer and second primary malignancies. Stockholm Breast Cancer Study Group. J Natl Cancer Inst.

[9] Deng H, Huang X, Fan J (2010). A variant of estrogen receptor-alpha, ER-alpha36 is expressed in human gastric cancer and is highly correlated with lymph node metastasis. Oncol Rep.

[10] Wang X, Deng H, Zou F (2013). ER-alpha36-mediated gastric cancer cell proliferation via the c-Src pathway. Oncol Lett.

[11] Wang Z, Zhang X, Shen P (2006). A variant of estrogen receptor-α, hER-α36: transduction of estrogen- and antiestrogen-dependent membrane-initiated mitogenic signaling. Proc Natl Acad Sci USA.

[12] Fu ZQ, Yang Y, Song J (2010). LiCl attenuates thapsigargin-induced tau hyperphosphorylation by inhibiting GSK-3beta in vivo and in vitro. J Alzheimers Dis.

[13] Deng H, Zhen H, Fu Z (2012). The antagonistic effect between STAT1 and Survivin and its clinical significance in gastric cancer. Oncol Lett.

[14] Kang L, Zhang X, Xie Y (2010). Involvement of estrogen receptor variant ER-alpha36, not GPR30, in nongenomic estrogen signaling. Mol Endocrinol.

[15] Suntharalingam A, Abisambra JF, O’Leary JC (2012). Glucose-regulated protein 94 triage of mutant myocilin through endoplasmic reticulum-associated degradation subverts a more efficient autophagic clearance mechanism. J Biol Chem.

[16] Zheng HC, Takahashi H, Li XH (2008). Overexpression of GRP78 and GRP94 are markers for aggressive behavior and poor prognosis in gastric carcinomas. Hum Pathol.

[17] Jones PF, Jakubowicz T, Pitossi FJ, Maurer F, Hemmings BA (1991). Molecular cloning and identification of a serine/threonine protein kinase of the second-messenger subfamily. Proc Natl Acad Sci USA.

[18] Garcia GE, Nicole A, Bhaskaran S (2006). Akt-and CREB-mediated prostate cancer cell proliferation inhibition by Nexrutine, a Phellodendron amurense extract. Neoplasia.

[19] Gottlob K, Majewski N, Kennedy S (2001). Inhibition of early apoptotic events by Akt/PKB is dependent on the first committed step of glycolysis and mitochondrial hexokinase. Genes Dev.

[20] Ang KL, Shi DL, Keong WW, Epstein RJ (2005). Upregulated Akt signaling adjacent to gastric cancers: implications for screening and chemoprevention. Cancer Lett.

[21] Oki E, Baba H, Tokunaga E (2005). Akt phosphorylation associates with LOH of PTEN and leads to chemoresistance for gastric cancer. Int J Cancer.

[22] Yu HG, Ai YW, Yu LL (2008). Phosphoinositide 3-kinase/Akt pathway plays an important role in chemoresistance of gastric cancer cells against etoposide and doxorubicin induced cell death. Int J Cancer.

[23] Guo RX, Wei LH, Tu Z (2006). 17 beta-estradiol activates PI3K/Akt signaling pathway by estrogen receptor (ER)-dependent and ER-independent mechanisms in endometrial cancer cells. J Steroid Biochem Mol Biol.

[24] Stoica GE, Franke TF, Wellstein A (2003). Estradiol rapidly activates Akt via the ErbB2 signaling pathway. Mol Endocrinol.

[25] Park S, Song J, Joe CO, Shin I (2008). Akt stabilizes estrogen receptor alpha with the concomitant reduction in its transcriptional activity. Cell Signal.

